# EETs Attenuate Ox-LDL-Induced LTB_4_ Production and Activity by Inhibiting p38 MAPK Phosphorylation and 5-LO/BLT1 Receptor Expression in Rat Pulmonary Arterial Endothelial Cells

**DOI:** 10.1371/journal.pone.0128278

**Published:** 2015-06-02

**Authors:** Jun-xia Jiang, Shui-juan Zhang, Yao-kang Xiong, Yong-liang Jia, Yan-hong Sun, Xi-xi Lin, Hui-juan Shen, Qiang-min Xie, Xiao-feng Yan

**Affiliations:** 1 The Second Affiliated Hospital, Zhejiang University School of Medicine, Hangzhou, Zhejiang, China; 2 Zhejiang Respiratory Drugs Research Laboratory of China FDA, Zhejiang University School of Medicine, Hangzhou, Zhejiang, China; 3 College of Pharmaceutical Science, Zhejiang Chinese Medical University, Hangzhou, Zhejiang, China; Faculty of Medicine & Health Sciences, UNITED ARAB EMIRATES

## Abstract

Cytochrome P-450 epoxygenase (EPOX)-derived epoxyeicosatrienoic acids (EETs), 5-lipoxygenase (5-LO), and leukotriene B_4_ (LTB_4_), the product of 5-LO, all play a pivotal role in the vascular inflammatory process. We have previously shown that EETs can alleviate oxidized low-density lipoprotein (ox-LDL)-induced endothelial inflammation in primary rat pulmonary artery endothelial cells (RPAECs). Here, we investigated whether ox-LDL can promote LTB_4_ production through the 5-LO pathway. We further explored how exogenous EETs influence ox-LDL-induced LTB_4_ production and activity. We found that treatment with ox-LDL increased the production of LTB_4_ and further led to the expression and release of both monocyte chemoattractant protein-1 (MCP-1/CCL2) and intercellular adhesion molecule-1 (ICAM-1). All of the above ox-LDL-induced changes were attenuated by the presence of 11,12-EET and 14,15-EET, as these molecules inhibited the 5-LO pathway. Furthermore, the LTB_4_ receptor 1 (BLT1 receptor) antagonist U75302 attenuated ox-LDL-induced ICAM-1 and MCP-1/CCL2 expression and production, whereas LY255283, a LTB_4_ receptor 2 (BLT2 receptor) antagonist, produced no such effects. Moreover, in RPAECs, we demonstrated that the increased expression of 5-LO and BLT1 following ox-LDL treatment resulted from the activation of nuclear factor-κB (NF-κB) via the p38 mitogen-activated protein kinase (MAPK) pathway. Our results indicated that EETs suppress ox-LDL-induced LTB_4_ production and subsequent inflammatory responses by downregulating the 5-LO/BLT1 receptor pathway, in which p38 MAPK phosphorylation activates NF-κB. These results suggest that the metabolism of arachidonic acid via the 5-LO and EPOX pathways may present a mutual constraint on the physiological regulation of vascular endothelial cells.

## Introduction

The biological features of cyclooxygenases (COXs) and lipoxygenases (LOXs) have been extensively studied, as their eicosanoid products play central roles in inflammatory processes. The LOX pathway is involved in the biosynthesis of hydroxyeicosatetraenoic acids (HETEs), lipoxins (LXs), and leukotrienes (LTs). These metabolites have been implicated in vasoregulatory and inflammatory events, such as asthma, allergic rhinitis, and atherosclerosis [1–3]. A growing body of evidence has shown that the LT pathway is critical to the development and progression of atherosclerotic lesions [4, 5]. LTs are potent lipid mediators that are derived from arachidonic acid (AA). The 5-lipoxygenase (5-LO) pathway is responsible for the production of leukotriene B_4_ (LTB_4_) and cysteinyl LTs (cysLTs). LTB_4_ is an extremely potent chemoattractant that promotes the adhesion of neutrophils, macrophages and other inflammatory cells to the vascular endothelium, thereby increasing vascular permeability. CysLTs can enhance the permeability and contractility of postcapillary venules [6]. LTB_4_-mediated effects are believed to occur through two G-protein coupled receptors (GPCRs): LTB_4_ receptor 1, or BLT1 (high affinity), and LTB_4_ receptor 2, BLT2 (low affinity) [7]. Increased expression of 5-LO in pulmonary artery endothelial cells (PAECs) has been reported in disease states such as primary pulmonary hypertension [8], chronic hypoxia [9] and antigen challenge [10]. Although the mechanism remains unclear, the induction of 5-LO expression may reflect endothelial dysfunction in the pulmonary vasculature, which has been found to be associated with the above diseases.

A third eicosanoid enzymatic pathway is the cytochrome P-450 epoxygenase (EPOX) pathway, which catalyzes two distinct enzymatic activities. EPOX hydroxylase enzymes generate HETEs that have cardiovascular and pro-inflammatory activities. Epoxyeicosatrienoic acids (EETs) that are derived from EPOX have multiple biological activities, including cardioprotection and anti-inflammatory properties [11–13]. The bioconversion of arachidonic acid (AA) into four EET regioisomers, 5,6-EET, 8,9-EET, 11,12-EET, and 14,15-EET, occurs via EPOX [14,15]. Rat CYP2C11 generates relatively equal proportions of 14,15-EET and 11,12-EET: 39% and 41%, respectively [16]. In human endothelial cells, 11,12-EET was found to significantly inhibit the expression of VCAM-1 in response to TNF-α, IL-1α, and LPS. By contrast, 14,15-EET had negligible effects, whereas 5,6-EET, 8,9-EET, and 11,12-DHET all led varying degrees of inhibition, but to a lesser extent than 11,12-EET. 11,12-EET also inhibited TNF-α-induced E-selectin and ICAM-1 expression [17]. Our previous studies have also shown that 11,12-EET and 14,15-EET can inhibit the oxidized low-density lipoprotein (ox-LDL)-induced expression of ICAM-1, MCP-1/CCL2 and E-selectin in rat pulmonary arterial endothelial cells (RPAECs) [18]. However, the exact mechanism of the suppressive effect of EETs on inflammation remains unclear.

Ox-LDL is associated with atherosclerotic events that involve the modulation of AA metabolism and the activation of inflammatory signaling. Lectin-like oxidized low-density lipoprotein receptor 1 (LOX-1) receptor acts as a cell surface receptor for ox-LDL on endothelial cells, and its expression is enhanced in proatherogenic settings [19, 20]. The LOX-1 receptor is upregulated by several stimuli, including ox-LDL, proinflammatory cytokines, endothelin-1, protein kinase-C, and angiotensin II [21]. We have previously demonstrated that EETs can induce protection against ox-LDL-induced endothelial dysfunction by blocking the binding of ox-LDL to the LOX-1 receptor, which subsequently decreases the expression of proinflammatory molecules [18].

In the present study, we found for the first time that ox-LDL can induce LTB_4_ production and activation in RPAECs. These increases in LTB_4_ production and activation can further induce the expression and release of ICAM-1 and MCP-1/CCL2 in RPAECs. Based on two lines of evidence, we speculated that LTB_4_ might mediate the atherosclerotic inflammatory response following ox-LDL treatment. These included the findings that LTB_4_ induces endothelium-dependent vascular responses [22] and that LTB_4_ may be an early mediator of atherosclerosis in patients presenting with obstructive sleep apnea [23, 24]. Indeed, LTB_4_ exerts a potent pro-inflammatory function via its interactions with cysLTs and BLT receptor subtypes, which are expressed in the inflammatory and structural cells that form the vascular wall. There has currently been a renewed interest in using LT antagonists for the treatment of atherosclerosis and its associated ischemic complications [25].

We have previously demonstrated that ox-LDL can mediate the release of the inflammatory factors ICAM-1 and MCP-1/CCL2 in RPAECs through activation of the MAPK/NF-κB pathway [18]. In this study, we found that the ox-LDL-induced LTB_4_ synthesis was also regulated by the same pathway. LTB_4_ synthesis regulated the expression and release of the pro-inflammatory factors ICAM-1 and MCP-1/CCL2 following ox-LDL stimulation. Therefore, we suggest that ox-LDL promotes LTB_4_ production by regulating the 5-LO pathway in endothelial cells. Following the above, we investigated the mechanisms behind the association of EETs and ox-LDL-induced LTB_4_ production.

## Materials and Methods

### 2.1 Ethics statement

All of the animal procedures and experiments conducted in this study were approved by the Ethics Committee of the Zhejiang University, School of Medicine. All animals received humane care, in accordance with the guide prepared by the Committee of Care and Use of Laboratory Animals of Zhejiang University (Permit No.ZJU201403-1-02-045). Male Sprague–Dawley rats (200–350 g, purchased from the Laboratory Animal Center, Zhejiang University; Ltd Certificate No. SCXK 2012–0002) were housed in groups of five and kept at 20–23°C under a 12/12 h light/dark cycle in 45–65% humidity. Standard laboratory food and water were provided *ad libitum*.

### 2.2. Reagents and antibodies

11,12-EET and 14,15-EET were purchased from Sigma-Aldrich (St. Louis, MO). Ox-LDL was purchased from Yiyuan Biotechnologies (Guangzhou, China) and is considered to be stable for six weeks after receipt when handled aseptically and stored at 2–8°C. Ox-LDL is a large protein (MW 3500 kDa) and has a diameter of 25.8 nm. It is composed of approximately 20–25% protein and 75–80% lipid. The lipid portion can be further described as being composed of 9% free cholesterol, 42% cholesteryl ester, 20–24% phospholipid, and 5% triglyceride. The ox-LDL that is produced by Yiyuan biotechnology is isolated from blood-bank-produced human plasma via ultra-centrifugation (1.019–1.063 g/cc). Following this, human LDL is oxidized using Cu_2_SO_4_ (oxidant) in PBS. Oxidation is terminated by adding an excess of EDTA-Na_2_. Each lot is analyzed via agarose gel electrophoresis for migration versus LDL. The lot of ox-LDL used in this study migrated 2.0-fold further than native LDL. TRIzol Reagent was purchased from Takara (Dalian, China). Primary antibodies against β-actin (Cell Signaling Technology, Danvers, MA), 5-LO and BLT1 (Santa Cruz Biotechnology, Santa Cruz, CA) were used in immunoblotting analysis. Rat LTB_4_, ICAM-1 and MCP-1/CCL2 enzyme-linked immunoassay (ELISA) kits were purchased from Boster (Wuhan, China). All other reagents and preparations were obtained as indicated.

### 2.3. Isolation and culture of RPAECs

Rat pulmonary artery endothelial cells (RPAECs) were isolated from pulmonary arteries as previously described with modification [26]. Briefly, male Sprague-Dawley rats (200–350 g) were sacrificed with 20% urethane. Following this, their hearts and lungs were excised. The mainstem pulmonary artery and two vessel generations were isolated from each heart, dissected, split, and fixed onto a 35 mm petri dish. The artery was inverted and the intimal lining was stacked on the dish. One week later, RPAECs were dissociated from the arteries. The cells were maintained in RPMI 1640 (HyClone, Logan, UT) that was supplemented with 10% fetal bovine serum (FBS) (HyClone) at 37°C in the presence of 5% CO_2_. Primary cells were allowed to grow and were passaged at confluency by trypsin digestion into culture flasks. RPAECs were characterized based on morphological criteria and by indirect immunofluorescence using an antibody specific to rat Factor VIII antigen; they were used up to passage 6.

### 2.4. RNA isolation and quantitative real-time PCR (qRT-PCR)

After the experimental treatment, total RNA was extracted from RPAECs using TRIzol Reagent (Takara, TaKaRa Biotechnology, Dalian, China) according to the manufacturer’s instructions. First-strand cDNA was generated from 4 μg of total RNA using oligo-dT to prime reverse transcription according to the manufacturer’s protocol. PCR primers were purchased from Shanghai Bioengineering Ltd (Shanghai, China). After an initial denaturation step of 10 min at 95°C, a two-step cycle procedure was used (denaturation at 95°C for 15 s, annealing and extension at 60°C for 1 min) for 40 cycles. Real-time PCR reactions were each performed in a total volume of 20 μL reaction mixture, containing 2 μL cDNA, 10.4 μL 2× SYBR Green 1 Master Mix (Takara), and 0.4 μL of each primer, using a Real-Time PCR System 7500 (Applied Biosystems). GAPDH was used as an internal control to normalize the samples. The transcript number was calculated using a 2^-ΔΔCt^ method (relative) [27]. The primers that were used in this experiment are indicated in Table 1.

**Table 1:** Primer sequences used in the present study.

Genes	Primer Sequences(5’-3’)
Rat ICAM-1	Sense: AGATCATACGGGTTTGGGCTTC
	Antisense: TATGACTCGTGAAAGAAATCAGCTC
Rat MCP-1/CCL2	Sense: ATGCAGGTCTCTGTCACGCT
	Antisense: GGTGCTGAAGTCCTTAGGGT
Rat 5-LO	Sense: CAAACCCCTGGAGAGAAGAAC
	Antisense: GCAATACCGAACACCTCAGAC
Rat BLT1	Sense: GCACCTGGAGTTTTGAAGTGA
	Antisense: TACGAACCTTTTGGGACACA
Rat GAPDH	Sense: CATGTTCGTCATGGGTGTGAACCA
	Antisense: ATGGCATGGACTGTGGTCATGAGT

### 2.5. Western blot analysis

After treatment, the cells were washed three times with ice-cold PBS and lysed in 100 μL radioimmunoprecipitation assay (RIPA) lysis buffer (Biyuntian Biotechnology, Haimen, China) containing 1 mM phenylmethylsulfonyl fluoride (PMSF) (Haoxin Biotechnology, Hangzhou, China). Protein concentration was measured using a BCA Protein Assay Kit (cwbiotech, Beijing, China). Cell lysates (30 μg) were separated by SDS-polyacrylamide gel electrophoresis and probed with antibodies specific for 5-LO, BLT1 (1:200, Santa Cruz, CA, USA) and β-actin (1:1000, Cell Signaling Technology, Beverly, MA, USA) overnight at 4°C; following this, they were probed with goat anti-rabbit 800 antibodies (1:5000) for 2 h at room temperature. Immunoreactive bands were visualized using a two-color infrared imaging system (Odyssey, LI-COR, USA).

### 2.6. Enzyme-linked immunosorbent assay (ELISA)

After treatment, supernatant was centrifuged, collected, and stored at -80°C prior to use. The presence of MCP-1/CCL2, ICAM-1 and LTB_4_ in the culture media of RPAECs was detected using ELISA kits (Boster, Wuhan, China); paired matched antibodies were used according to the manufacturer's instructions. Color absorbance at 450 nm was determined using a Bio-Rad microplate reader.

### 2.7. Statistical analysis

Data were presented as the mean ± S.E.M. GraphPad Prism V5.0 software was used for statistical analysis. Statistical tests were performed using SPSS software (version 16.0; SPSS, Chicago, IL). Differences between mean values of multiple groups were analyzed by either one-way analysis of variance (ANOVA) or Student's t test. Statistical significance was accepted at *P* < 0.05.

## Results

### 3.1. Ox-LDL triggers inflammatory responses and upregulates the expression and activity of the 5-LO pathway in rat pulmonary arterial endothelial cells

**Fig 1:**
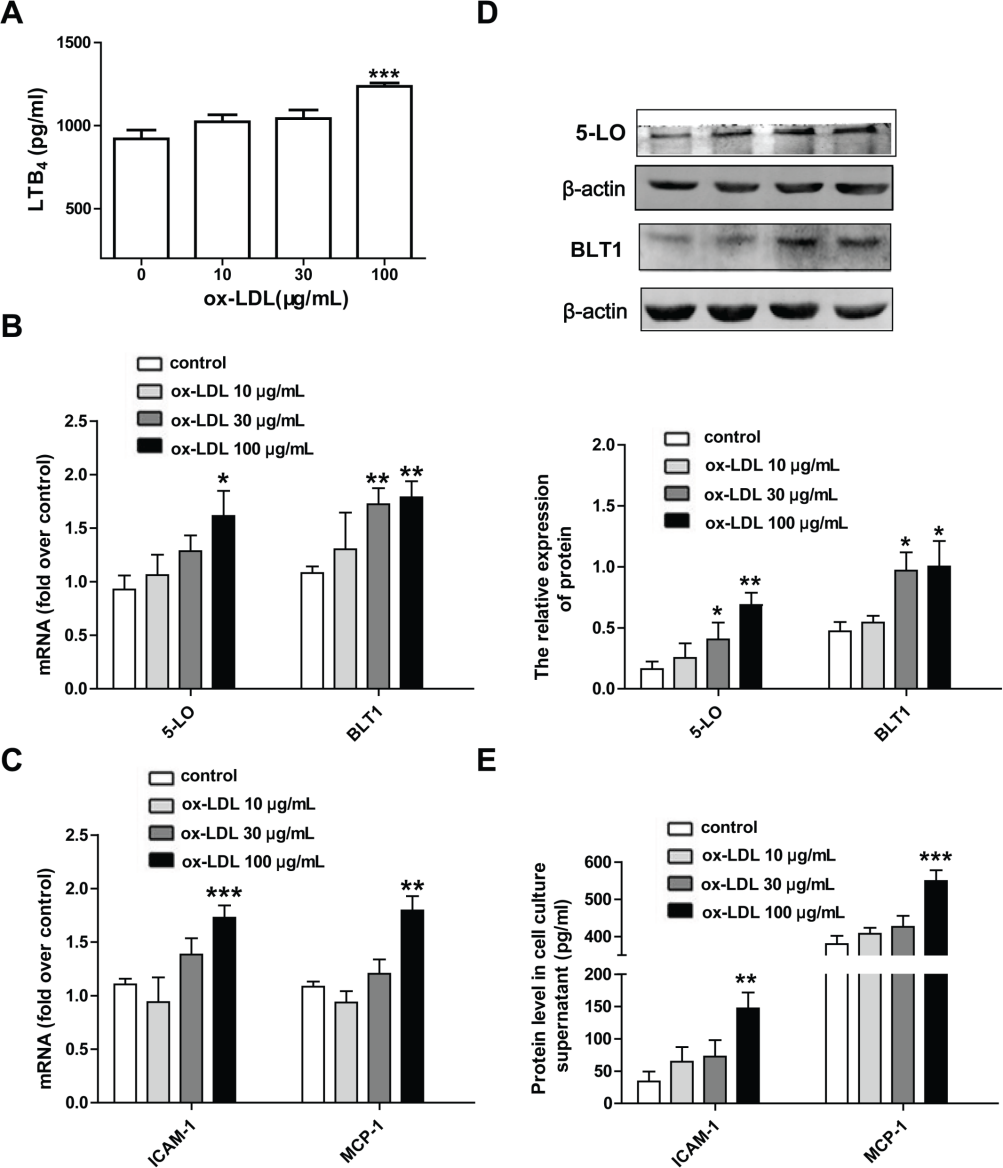
Ox-LDL induces the expression and release of ICAM-1 and MCP-1/CCL2 and up-regulates the 5-LO pathway. (A) RPAECs were exposed to different concentration of ox-LDL (10–100 μg/mL) for 24 h. LTB_4_ protein levels in cell culture supernatant was quantified by ELISA. (B, D) 5-LO and BLT1 mRNA and protein levels were respectively measured by qRT-PCR and western blot. (C, E) ICAM-1, MCP-1/CCL2 mRNA and protein levels in cell culture supernatant were quantified by qRT-PCR and ELISA. The data shown are means ± S.E.M. of results from at least four independent experiments. *P<0.05, **P<0.01, ***P<0.001 compared with the control group.

To determine whether the 5-LO pathway is involved in ox-LDL-induced endothelial inflammation, cultured RPAECs were treated with ox-LDL (10–100 μg/mL). Following this, the expression and release of the pro-inflammatory factors ICAM-1 and MCP-1/CCL2 were measured. We found that ox-LDL induced a marked elevation of ICAM-1 and MCP-1/CCL2 expression and release in a concentration-dependent manner in RPAECs (Fig 1C and 1E). Furthermore, 24 hours of stimulation with ox-LDL increased the levels of 5-LO and BLT1 mRNAs and proteins in RPAECs in a concentration-dependent manner (Fig 1B and 1D). Ox-LDL (100 μg/mL) also significantly increased the levels of the 5-LO metabolite LTB_4_ level in culture supernatants compared to non-stimulated control (*P*<0.001) (Fig 1A).

### 3.2. EETs inhibit ox-LDL-induced LTB_4_, ICAM-1 and MCP-1/CCL2 expression and secretion by suppressing the 5-LO pathway

**Fig 2:**
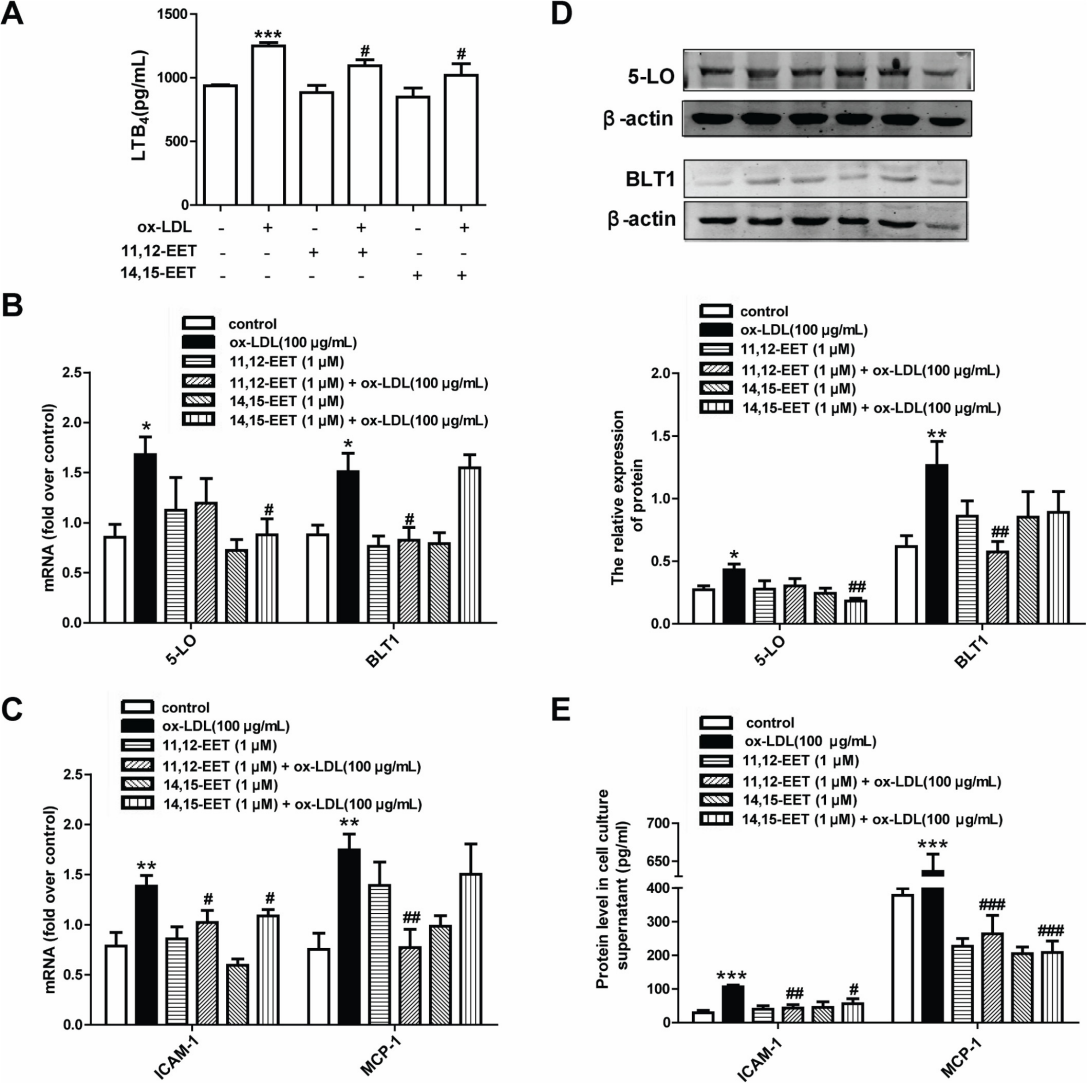
EETs inhibit ox-LDL-induced expression and production of ICAM-1 and MCP-1/CCL2 by down-regulating the 5-LO pathway in RPAECs. (A) RPAECs were incubated with the indicated concentrations of 11,12-EET or 14,15-EET (1 μM) for 30 min and then stimulated with ox-LDL (100 μg/mL) for 24 h. LTB_4_ protein levels in cell culture supernatant were quantified by ELISA. (B, D) 5-LO and BLT1 mRNA and protein levels were respectively determined by qRT-PCR and western blot. (C, E) ICAM-1 and MCP-1/CCL2 mRNA expression and protein levels in cell culture supernatant were quantified by qRT-PCR and ELISA. The data represent means ± S.E.M. from six independent experiments. *P<0.05, **P<0.01, ***P<0.001 compared with the control group. ^*#*^ *P*<0.05 compared with the ox-LDL (100 μg/mL)-treated group.

To confirm whether EETs affect the expression and activity of the 5-LO pathway, RPAECs were treated with 11,12-EET and 14,15-EET for 30 min and then stimulated with ox-LDL (100 μg/mL) for 24 h. ELISA was performed to assess the levels of the pro-inflammatory factors LTB_4_, ICAM-1 and MCP-1/CCL2. The levels of 5-LO and BLT1 proteins and mRNAs were examined by western blot analysis and real-time PCR. Pre-incubation with 11,12-EET or 14,15-EET at 1 μM significantly suppressed the ox-LDL stimulation induced expression of LTB_4_, ICAM-1 and MCP-1/CCL2 mRNAs and proteins in RPAECs (Fig 2A, 2C and 2E). We next examined the influence of EETs on the ox-LDL-induced expression of 5-LO and the BLT1 LTB_4_ receptor. As shown in Fig 2B and 2D, the presence of 14,15-EET dramatically decreased 5-LO mRNA and protein expression, whereas 11,12-EET decreased BLT1 expression.

### 3.3. LTB_4_ increases the expression and release of the pro-inflammatory factors ICAM-1 and MCP-1/CCL2

**Fig 3:**
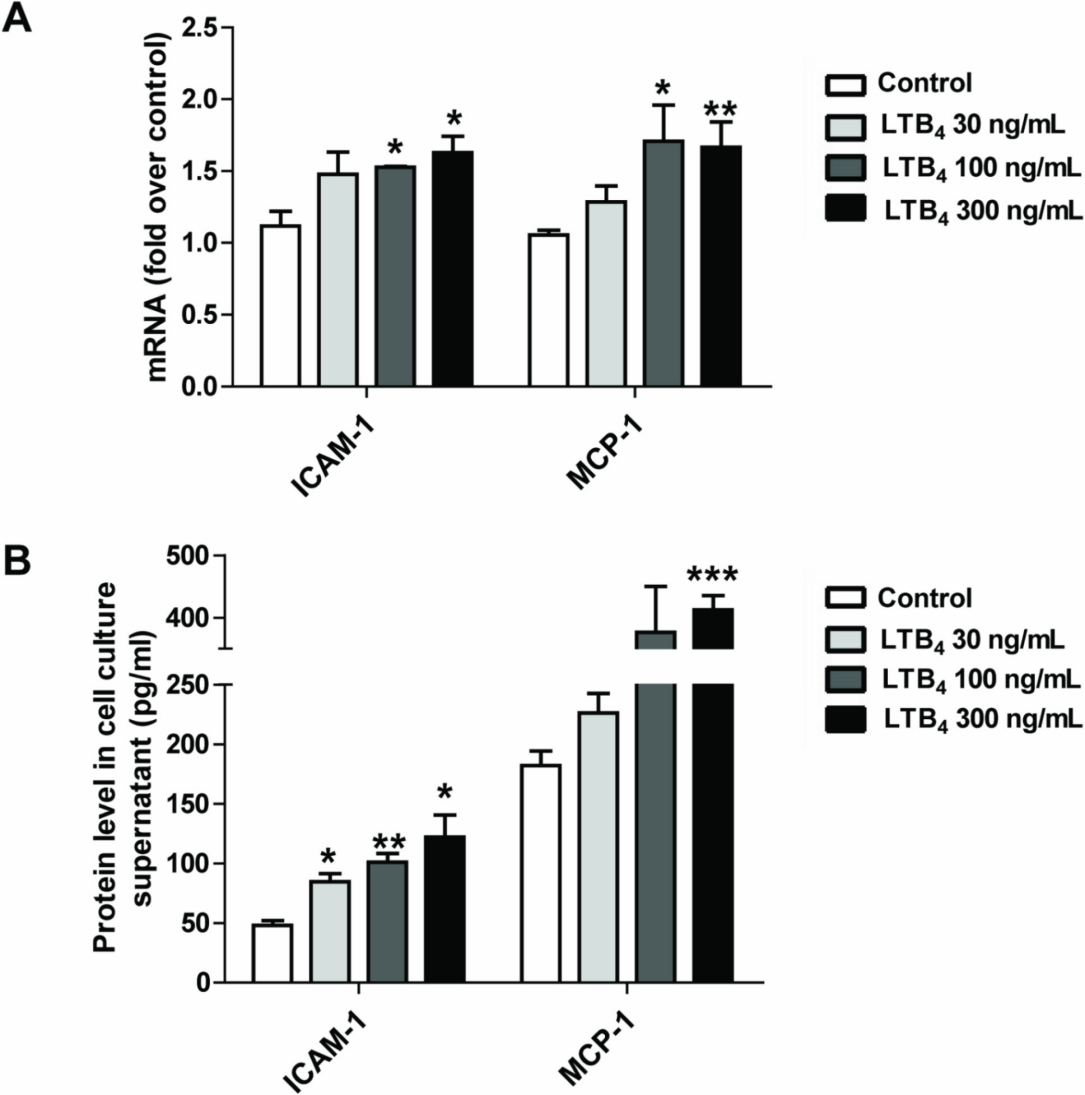
LTB_4_ induces ICAM-1 and MCP-1/CCL2 expression and release in rat pulmonary arterial endothelial cells. RPAECs were exposed to different concentrations of LTB_4_ (30–300 ng/mL) for 24 h. ICAM-1 and MCP-1/CCL2 mRNA and protein levels were respectively measured by qRT-PCR (A) and ELISA (B). The data represent means ± S.E.M. from six independent experiments. * P <0.05, ** P <0.01, *** P <0.001 compared with the control group.

LTB_4_ plays an important role in inflammatory responses during endothelial dysfunction. A previous report indicated that LTB_4_ might be an early mediator of atherosclerosis [23]. To determine whether LTB_4_ can regulate the expression and production of pro-inflammatory factors, primary RPAECs were treated with LTB_4_ (30–300 ng/mL), and levels of MCP-1/CCL2, a chemokine, and ICAM-1, an adhesion molecule, were measured. We found that treatment with 100–300 ng/mL of LTB_4_ robustly increased the expression of ICAM-1 and MCP-1/CCL2 mRNAs in RPAECs in a concentration-dependent manner (Fig 3A). We further confirmed these results by measuring ICAM-1 and MCP-1 /CCL2 release, and we found that 24 hours of LTB_4_ treatment augmented the secretion of these molecules into the culture media of RPAECs in a concentration-dependent manner (Fig 3B).

### 3.4. Role of LTB_4_ in ox-LDL-induced ICAM-1 and MCP-1/CCL2 expression

LTB_4_ exerts biological function through two receptors: BLT1 and BLT2 [4]. To determine whether ox-LDL-induced ICAM-1 and MCP-1/CCL2 expression is mediated by LTB_4_ receptors, primary RPAECs were treated with LTB_4_ receptor antagonists in the presence or absence of ox-LDL. ICAM-1 and MCP-1/CCL2 levels in supernatant were determined by ELISA.

**Fig 4:**
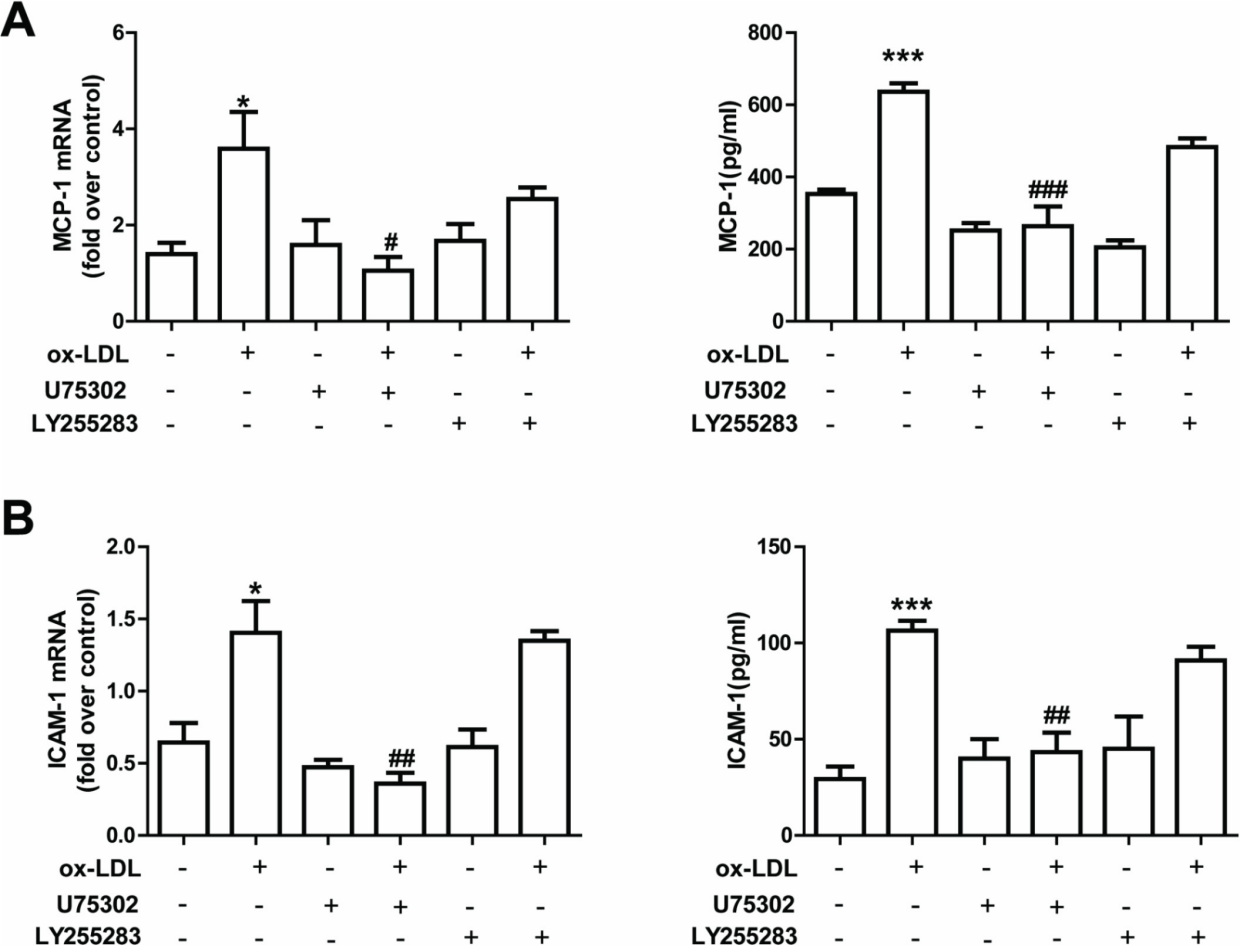
Role of LTB_4_ receptor antagonists on ICAM-1 and MCP-1/CCL2 expression in RPAECs. Cells were pretreated with a BLT1 receptor antagonist (U75302) (10 μM) and a BLT2 receptor antagonist (LY255283) (10 μM) and then stimulated with ox-LDL (100 μg/mL) for 24 h. Expression levels of MCP-1/CCL2 (A) and ICAM-1 (B) mRNAs and proteins were determined by qRT-PCR and ELISA, respectively. The data represent means ± S.E.M. from five independent experiments. * P <0.05, ** P <0.01, *** P <0.001 compared with the control group. ^*#*^ *P* <0.05 and ^*##*^ *P* <0.01 compared with the ox-LDL (100 μg/mL)-stimulated group.

At 24 hours after treatment with LTB_4_, we found a concentration-dependent increase in ICAM-1 and MCP-1/CCL2 expression and release (Fig 3A and 3B). Furthermore, pretreatment using a BLT1 receptor antagonist, U75302, inhibited ox-LDL-induced ICAM-1 and MCP-1/CCL2 production (Fig 4A and 4B). However, the pretreatment using a BLT2 receptor antagonist, LY255283, did not produce the same effects (Fig 4A and 4B). These results indicate that one way in which ox-LDL induces inflammatory responses in RPAECs is by activating the LTB_4_/BLT1 receptor.

### 3.5. Pharmacological suppression of the p38 MAPK and NF-κB pathways inhibits 5-LO and BLT1 expression in RPAECs

To assess the potential molecular mechanisms behind ox-LDL-mediated 5-LO pathway activation, we examined how using inhibitors of the 5-LO pathway affected the expression of 5-LO and BLT1 mRNAs and proteins in RPAECs. After 30 minutes of exposure to a p38 inhibitor (SB203580) and an NF-κB inhibitor (PDTC), we found marked reductions in 5-LO and BLT1 mRNA and protein levels, which had been previously upregulated following treatment with ox-LDL. Thus, these data suggest that ox-LDL stimulation upregulates 5-LO and BLT1 expression by activating p38 MAPK and NF-κB signaling pathways.

## Discussion

The current study is the first to describe the cellular mechanisms that are responsible for the inhibitory effects of epoxygenase-derived eicosanoids, including 11,12-EET and 14,15-EET, on ox-LDL-mediated pro-inflammatory mediators (ICAM-1 and MCP-1/CCL2) in RPAECs. Leukotrienes have long been described as lipid mediators and were initially shown to have significant effects on the pathogenesis of allergic rhinitis and bronchial asthma. Recent research has revealed a new role for leukotrienes in the vascular inflammation that is associated with atherosclerotic vascular disease [28, 29]. As noted, LTB_4_, a 5-LO-derived eicosanoid, is a prominent pro-inflammatory and chemotactic mediator and can regulate the production of many additional inflammatory mediators by activating nuclear signals [30]. In an earlier exploratory study, we tested the effects of low-density lipoprotein (LDL) on LTB_4_ production, ICAM-1 and MCP-1/CCL2 expression in RPAECs. We found ox-LDL is more potent, and LDL does not induce significant inflammatory responses in RPAECs (S2 Fig). Therefore, we selected ox-LDL stimulation as the model in our current study. In this study, we showed that ox-LDL promoted LTB_4_ production in RPAECs (Fig 1A). We used hydrogen peroxide (H_2_O_2_) as a control to determine whether our results were due to an oxidant effect or an ox-LDL-specific effect. We examined the expression and release of ICAM-1 and MCP-1/CCL2 and the production of LTB_4_ following H_2_O_2_ stimulaiton in rat pulmonary artery endothelial cells. H_2_O_2_ (125–500 μM)-induced inflammatory responses were not found to be statistically significant (S3 Fig). Therefore, it is a specific effect induced by ox-LDL. We then demonstrated that 1 μM concentrations of 11,12-EET and 14,15-EET (Fig 2A) decreased ox-LDL-induced LTB_4_ production in RPAECs, which represents the first description that EETs can affect the ox-LDL-stimulated production of LTB_4_ in pulmonary arterial endothelial cells.

Although the exact mechanism behind the suppressive effects of EETs on LTB_4_ production remains unclear, the induction of LTB_4_ production in RPAECs revealed that ox-LDL has a potential role in initiating inflammation through the 5-LO pathway. Additional observations suggested that EETs might directly inhibit 5-LO activity. Liu et al. [31] demonstrated that soluble epoxide hydrolase (sEH) inhibition stabilizes CYP450 production of EETs and that inhibition of sEH enhances the anti-inflammatory effects that are produced by a protein inhibitor of 5-lipoxygenase activation in a murine model. Revermann et al. [32] reported that a pirinixic acid derivative, LP105, is a potent inhibitor of monocyte 5-LO and can reduce AngII-induced vascular remodeling in mice. Our results regarding the anti-inflammatory effects of 11,12-EET and 14,15-EET in RPAECs are consistent with these findings. Pretreatment with 11,12-EET or 14,15-EET at 1 μM causes significant suppression of 5-LO and its metabolite LTB_4_, both of which are increased by ox-LDL stimulation (Fig 2A, 2B and 2D). These results suggest that a beneficial effect is imparted by the increased formation of protective arachidonic acid derivatives, particularly with respect to EETs.

It has been reported that LTB_4_-BLT signaling is involved in atherogenesis [33–35]. In early atherogenesis, the 5-LO metabolite LTB_4_ enhances inflammatory responses by several mechanisms, one of which is the induction of pro-inflammatory mediator release and foam cell formation that is orchestrated by BLT receptors [5]. It has been reported that symptomatic atherosclerotic plaques express elevated levels of both 5-LO and LTB_4_. This evidence indicates that LTB_4_ might be a key mediator of 5-LO-dependent plaque instability [36]. In line with these reports, we showed here that the inhibition of BLT1 attenuated the ox-LDL stimulation induced upregulation of ICAM-1 and MCP-1/CCL2 expression in RPAECs (Fig 4). Moreover, LTB_4_ activation is capable of stimulating the production of ICAM-1 and MCP-1/CCL2 in RPAECs (Fig 3A and 3B), suggesting that LTB_4_ is a major activator of inflammation. In a study [37] that compared healthy subjects with patients presenting with carotid atherosclerosis, a significant increase was found in the expression of all of the components of the 5-LO pathway, in addition to levels of BLT1 and BLT2 mRNA (measured by real-time PCR), in peripheral blood mononuclear cells; levels of LTB_4_ were also increased in plasma (measured by ELISA). However, whether ox-LDL promotes BLT1 and BLT2 expression has not yet been investigated. In our study, we found that expression of the BLT1 LTB_4_ receptor was decreased by treatment with 11,12-EET (Fig 2B, Fig 2D). These results suggest that the LTB_4_/BLT1 receptor pathway plays a role in generating the anti-inflammatory effects that are produced by EETs in ox-LDL-stimulated RPAECs. BLT1 expression was decreased by 11,12-EET, whereas 14,15-EET dramatically decreased 5- LO expression. The differential action of the two EETs in mediator production and 5-LO pathway expression was due to the differences in chemical structure, which can significantly impact their effects on vascular activity. These phenomena has been previously reported that 11,12-EET significantly attenuates the TNF-α-stimulated endothelial activation and leukocyte adhesion by inhibiting IκBα degradation and NF-κB activation, whereas 14,15-EET results in a lower activation of NF-κB [38]. Another study suggests that EETs may act in a cell type-specific manner [39]. These results may explain the differential actions of the two EETs in the present study.

**Fig 5:**
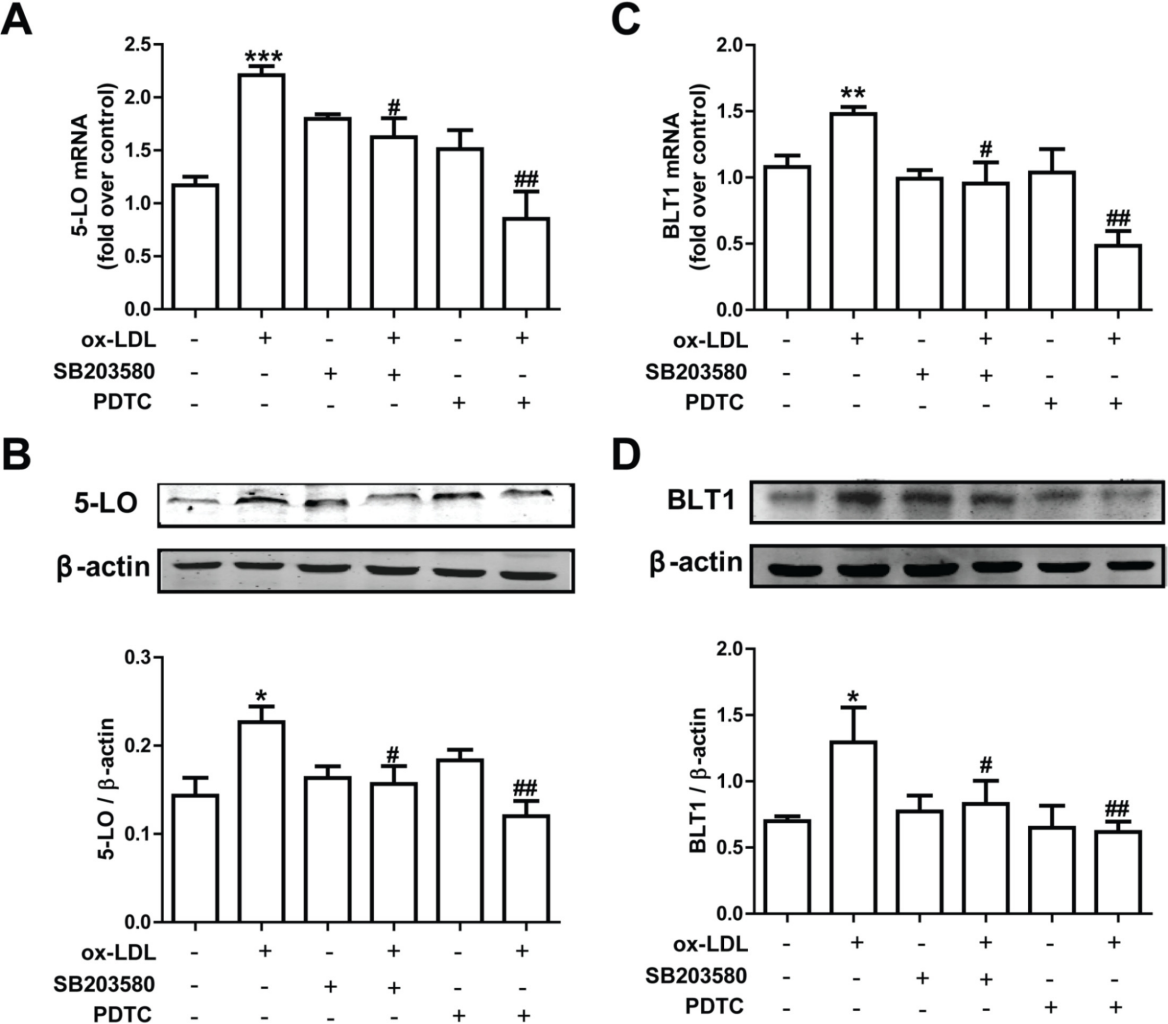
The p38 MAPK and NF-κB signaling pathways are involved in ox-LDL-mediated expression of 5-LO and BLT1. RPAECs were pre-treated with a p38 inhibitor (10 μM SB203580) or an NF-κB inhibitor (20 μM PDTC) for 30 min and then incubated with ox-LDL (100 μg/mL) for another 24 h. The levels of 5-LO and BLT1 mRNAs and proteins were determined by qRT-PCR (A, C) and western blot (B, D), respectively. The data represent the means ± S.E.M. from four independent experiments. * P <0.05 and ** P <0.01 compared with the untreated group. ^*#*^ *P* <0.05 and ^*##*^ *P* <0.01 compared with the ox-LDL (100 μg/mL)-stimulated group.

As a principle enzyme of leukotriene production, 5-LO is regulated by Ca^2+^, ox-LDL, ROS, and HNE in the NF-κB/ERK and Sp1/p38 MAPK pathways [40, 41]. In this study, we found that ox-LDL promoted the expression of 5-LO and BLT1 mRNAs and proteins through the p38 MAPK/ NF-κB pathway in rat PAECs. Results from qRT-PCR and western blotting indicated that ox-LDL promotes the expression of 5-LO and BLT1 mRNAs and proteins in a concentration-dependent manner. The maximum effect produced by ox-LDL on 5-LO expression was at a concentration of 100 μg/mL: the same concentration at which the p38 MAPK and NF-κB pathways became the most activated in a previously reported study [18]. To determine whether the p38 MAPK and NF-κB pathways were involved in the ox-LDL-induced expression of 5-LO and BLT1, a p38 MAPK inhibitor, SB203580, and an NF-κB inhibitor, PDTC, were employed. Following treatment with these inhibitors, Ox-LDL-induced increases to the expression of 5-LO and BLT1 mRNAs and proteins were significantly inhibited (Fig 5). Furthermore, our earlier data also demonstrated that EETs suppress ox-LDL-induced p38 phosphorylation in RPAECs [18]. This phosphorylation of p38 MAPK might modulate the nuclear translocation of various transcription factors, including NF-κB [42]. These data support that the p38 MAPK and NF-κB signal transduction pathways play a role in ox-LDL-regulated 5-LO and BLT1 expression. Our results demonstrate that 5-LO and BLT1 expression is regulated by ox-LDL-induced NF-κB activation through the p38 MAPK pathway. Because 5-LO is a crucial factor in leukotriene production, inhibition of this pathway may represent a potential therapeutic target for treating vascular endothelium mediated inflammatory responses in patients with cardiovascular disease. With the rising number of indications for anti-LT therapy, drug development strategies centered on 5-LO inhibition are becoming increasingly popular. The most advanced drug that has been developed thus far is an N-hydroxyurea derivative known as Atreleuton. Atreleuton was recently examined in a phase II study conducted on atherosclerotic patients with established cardiovascular disease following acute coronary syndrome. The results of this trial demonstrated that Atreleuton potently reduces leukotriene production and may also affect the formation of atherosclerotic plaques [43]. Another study also revealed that the Cys-LT1 receptor antagonist Montelukast might be beneficial toward the secondary prevention of cardiovascular disease [44].

## Conclusion

In conclusion, the results of the present study confirmed the following: (1) Ox-LDL promotes the production of LTB_4_, ICAM-1 and MCP-1/CCL2 in conditions of endothelial inflammation by up-regulating 5-LO expression through p38 MAPK/NF-κB activation; (2) EETs produce anti-inflammatory effects by inhibiting LTB_4_, ICAM-1 and MCP-1/CCL2 release in RPAECs through the 5-LO pathway; (3) the LTB_4_/BLT1 receptor pathway is involved in the production of EET-mediated anti-inflammatory effects. We proposed a potential mechanism to explain the protective effects of EETs on endothelial dysfunction in RPAECs.

## Supporting Information

S1 Fig Ox-LDL increases cell viability in RPAECs. (TIF)

S2 Fig The effects of LDL on the expression and release of ICAM-1, MCP-1/CCL2 and the 5-LO pathway. (TIF)

S3 Fig The effects of H_2_O_2_ on the expression and release of ICAM-1 and MCP-1/CCL2 and LTB_4_ release. (TIF)

S4 Fig EETs inhibit ox-LDL-induced activation of MAPK and NF-κB in RPAECs. (TIF)
