# Supplementary figures and images for: EETs Attenuate Ox-LDL-Induced LTB4 Production and Activity by Inhibiting p38 MAPK Phosphorylation and 5-LO/BLT1 Receptor Expression in Rat Pulmonary Arterial Endothelial Cells
Source: PLoS One. 2015 Jun 2;10(6):e0128278. doi: 10.1371/journal.pone.0128278 (PMC4452698; doi:10.1371/journal.pone.0128278)

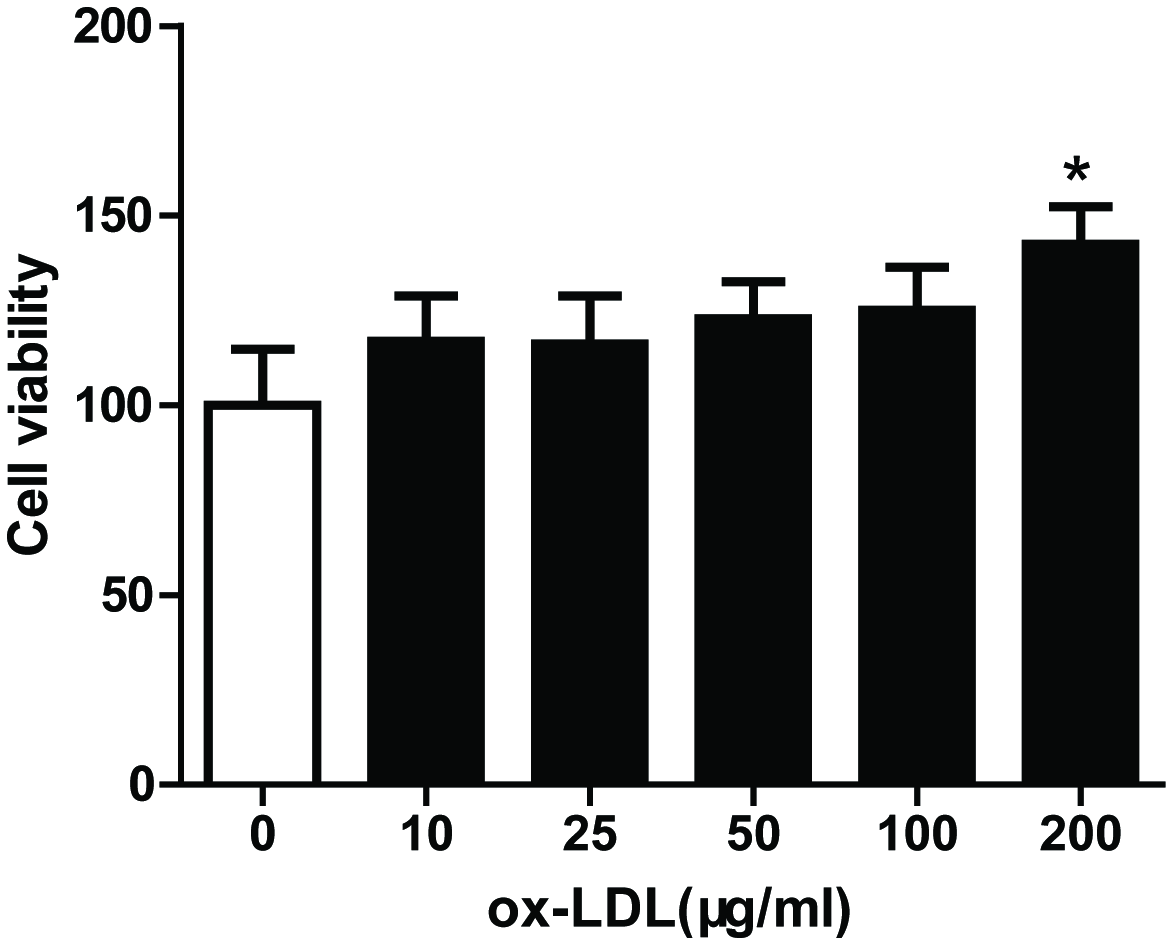

Supplement: S1 Fig — (TIF) [file pone.0128278.s001.tif]

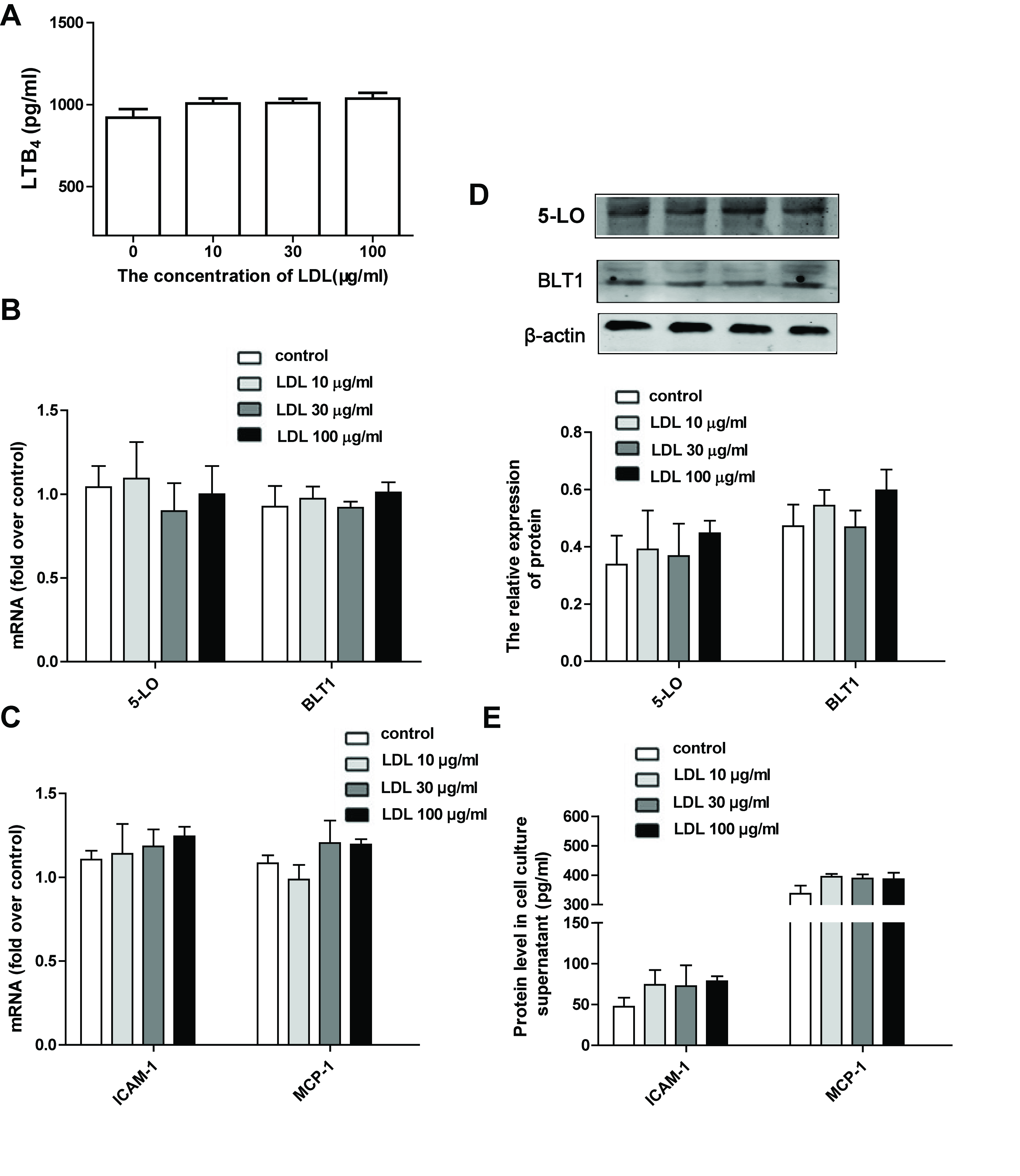

Supplement: S2 Fig — (TIF) [file pone.0128278.s002.tif]

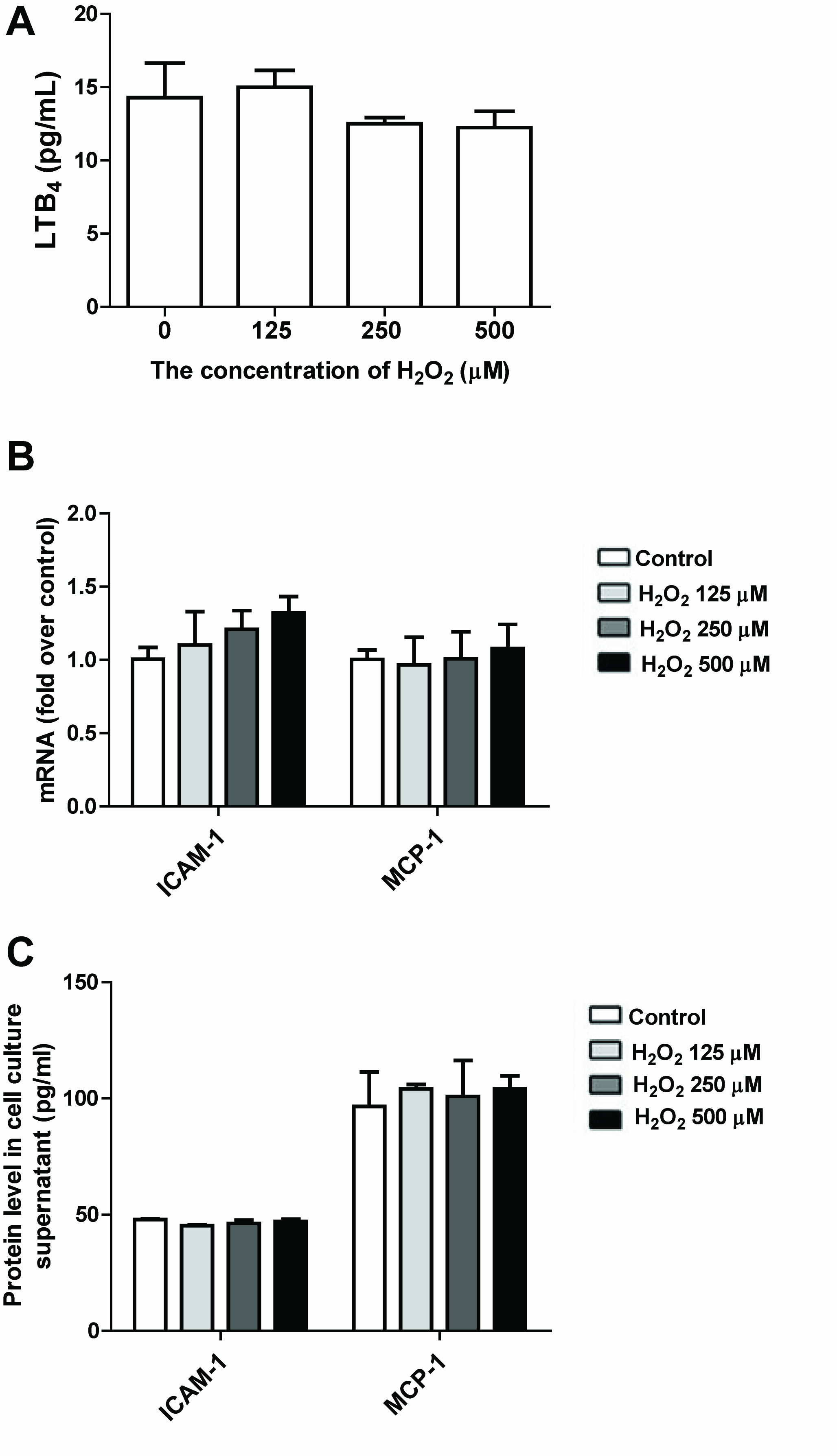

Supplement: S3 Fig — (TIF) [file pone.0128278.s003.tif]

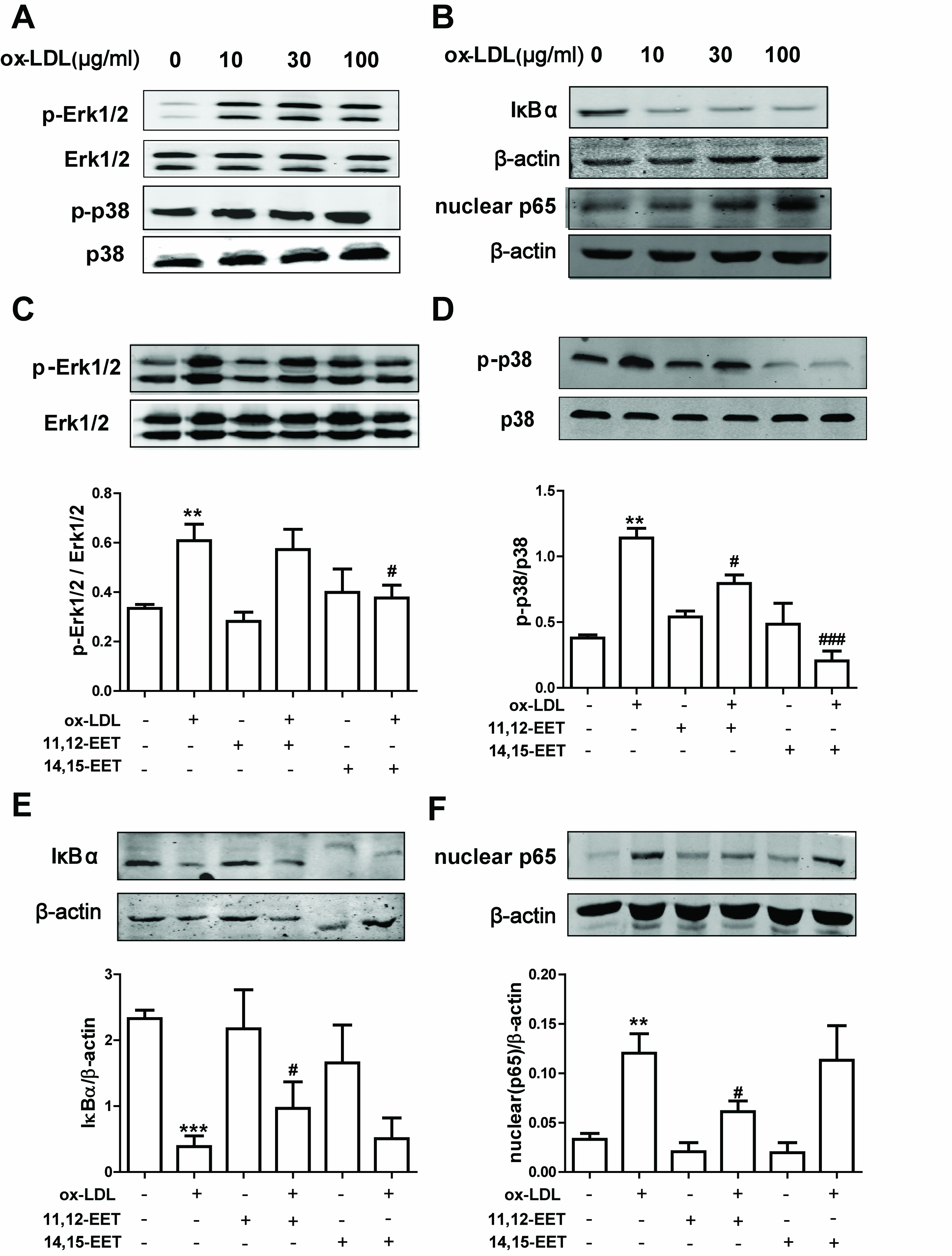

Supplement: S4 Fig — (TIF) [file pone.0128278.s004.tif]
